# Head-to-Head Comparison of the Expression Differences of NECTIN-4, TROP-2, and HER2 in Urothelial Carcinoma and Its Histologic Variants

**DOI:** 10.3389/fonc.2022.858865

**Published:** 2022-04-19

**Authors:** Yu Fan, Qinhan Li, Qi Shen, Zhifu Liu, Zhenan Zhang, Shuai Hu, Wei Yu, Zhisong He, Qun He, Qian Zhang

**Affiliations:** Department of Urology, Peking University First Hospital, Institute of Urology, National Research Center for Genitourinary Oncology, Peking University, Beijing, China

**Keywords:** urothelial carcinoma, ADC, nectin-4, Trop-2, HER2, histologic variants

## Abstract

**Background:**

Antibody–drug conjugates (ADC), such as enfortumab vedotin (EV), sacituzumab govitecan (SG), and RC-48, have shown outstanding response rates to local advanced or metastatic urothelial carcinoma (UC). However, their corresponding target expression characteristics in UC and its histologic variants were unknown.

**Methods:**

We detected the expression of NECTIN-4, TROP-2, and HER2, which are the corresponding targets of ADCs EV, SG, and RC-48 in muscle-invasive UC through immunohistochemistry.

**Results:**

161 consecutive samples from 2017 to 2021 of muscle-invasive UC and its histologic variants were obtained in Peking University First Hospital. Variant histology types included 72UC, 10 squamous carcinomas, 23 glandular carcinomas, 19 small cell carcinomas, 19 micropapillary variants, and 18 nested variants. NECTIN-4 expression was found to be 57/72 (79.2%), 10/10 (100%), 15/23 (65.2%), 4/19 (21.1%), 15/19 (78.9%), and 16/18 (88.9%) in conventional UC, squamous carcinoma, glandular carcinoma, small cell carcinoma, micropapillary, and nested variant, respectively, compared with 65/72 (90.3%), 8/10 (80.0%), 13/23 (56.5%), 3/19 (15.8%), 16/19 (84.2%), and 15/18 (83.3%) of TROP-2, and 26/72 (36.1%), 0, 5/23 (21.7%), 6/19 (31.6%), 5/19 (26.3%), and 7/18 (38.9%) of HER2.

## Introduction

Urothelial carcinoma (UC) is the second most common genitourinary tract cancer, affecting >80,000 new patients and causing >17,000 deaths every year in the United States (1). UC commonly arises from the urinary bladder but also involves the renal pelvis, the ureter, or the urethra. Conventional UC is the most common histologic type and accounts for around 90% of all UC, and the remaining 10% show different histologic variants such as squamous carcinoma, glandular carcinoma, small cell carcinoma, micropapillary variant, and nested variant (2, 3). Squamous carcinoma is the most common subtype, accounting for 3%–5% of all UC, followed by glandular carcinoma of 1.5% and small cell carcinoma of 0.7% (4). Furthermore, Chinese people are reported to have different clinicopathological characteristics and oncologic outcomes of UC in the United States, with more adverse pathological features (5). However, despite multiple histologic subtypes, UC has been managed similarly. For advanced or metastatic UC, cisplatin-based chemotherapy is the first-line therapy due to its high response rate. For the cisplatin-ineligible patients, carboplatin-based chemotherapy combined with an immune checkpoint inhibitor (ICI) is recommended (6). Recently, antibody–drug conjugates (ADC), the emerging agents that combine a cytotoxic agent with a monoclonal antibody (mAb) as a delivery molecule, have been promising as the new treatment approach for advanced or metastatic UC (7).

The study about ADCs could be dated from the late 1950s, when polyclonal and murine monoclonal antibodies were detected preclinically with conjugates comprising radionuclides, toxin, and drugs (8). However, these first-generation ADCs suffered from immune responses to the xenogeneic antibodies, limiting their clinical application. Recently, second- and third-generation ADCs such as enfortumab vedotin (EV), sacituzumab govitecan (SG), and RC-48, using monoclonal mAbs with better-defined precision targets, combined with more toxic payloads have emerged as a new line of approved ADCs. Enfortumab vedotin (EV), a novel ADC composed of an anti-NECTIN-4 antibody with the microtubule-disrupting cytotoxic agent monomethyl auristatin E (MMAE), binds to cells that express NECTIN-4, a cell adhesion molecule highly expressed in many solid tumors including UC. Then, MMAE is internalized and released into the target cells and impairs the formation of the microtubule network (9, 10). TROP-2 is a transmembrane glycoprotein overexpressed in many solid tumors, including UC, and linked with worse prognosis (11, 12). Sacituzumab govitecan (SG) is an ADC composed of SN-38 conjugated to an anti-Trop-2-humanized mAb, resulting in double-stranded DNA breaks during the mitotic S phase of affected cells (13). HER2 is a growth-promoting tyrosine kinase receptor, whose overexpression, though uncommonly, is highly associated with tumor progression and poor prognosis in UC (14, 15).RC48-ADC is a novel humanized anti-HER2 antibody conjugated with MMAE *via* a cleavable linker, impairing the formation of the microtubule network of target cells (16).

During the immunotherapy era, the PD-L1 expression situation was proven to be an important prognostic factor in both bladder cancer and upper tract urothelial carcinoma undergoing immunotherapy (17–19). Although it is still unknown whether a high expression of the ADC-corresponding targets is linked to a better efficacy, the expression of these proteins is believed to be essential for the response to ADC as it is the port of entry to tumor cells. The expression of NECTIN-4 in the muscle-invasive UC is reported to be 68.2% (20), compared with 8.7% of HER2 (14), and TROP-2 is known to be expressed in normal urothelium and in ≤83% of urothelial carcinoma (21). However, it is unknown whether the protein expression is related to the clinicopathologic features of the patient. The head-to-head comparison regarding the expression differences of these targets in UC and its histologic variants is rare, which could have potential implications in therapeutic strategies. In the present study, we conducted a head-to-head comparison of expression differences of NECTIN-4, TROP-2, and HER2 in muscle-invasive UC and its histologic variants, discussing the possible tendency of ADC choice in different pathologic subtypes of UC.

## Materials and Methods

161 consecutive samples from 2017 to 2021 of muscle-invasive UC and its histologic divergent types were obtained from the patients who underwent radical cystectomy and radical nephroureterectomy without adjuvant or neoadjuvant therapy before in the Department of Urology, Peking University First Hospital. Variant histology types included 10 squamous carcinomas, 23 glandular carcinomas, 19 small cell carcinomas, 19 micropapillary variants, and 18 nested variants. The histopathology of tumors was graded according to the World Health Organization histologic grading system and staged according to the TNM staging system (22, 23). The slides were reviewed by 3 expert urologic pathologists (QH, QS, and SH), and a representative section was chosen and recut to perform immunohistochemical stains. The study was approved by the ethics committee of Peking University First Hospital.

Moreover, the samples with muscle-invasive bladder cancer (MIBC) of conventional pathological type were grouped into luminal and basal/squamous subtypes based on expressions of GATA3 and KRT5/6 through immunohistochemistry (24, 25). Tissues that were KRT5/6-positive and GATA3-negative were considered of basal-like phenotype, while tissues that were GATA3-positive were deemed of luminal-like phenotype. KRT5/6-positive and GATA3-positive were defined as KRT5/6 2+/3+ and GATA3 2+/3+, respectively.

### Immunohistochemistry

The expressions of NECTIN-4, TROP-2, and HER2 were evaluated according to standard immunohistochemistry protocols. Briefly, 4-μm-thick sections from formalin-fixed paraffin-embedded specimens were deparaffinized in xylene, rehydrated in decreasing concentrations of ethanol, and washed in distilled water. Following antigen retrieval with Tris–EDTA buffer, endogenous peroxidase blocking with 3% hydrogen peroxidase was performed. Sections were incubated with 10% normal blocking serum in Tris-buffered saline at room temperature for 20 min. The commercially available primary antibodies used in this study were anti-human NECTIN-4, TROP-2, and HER2 rabbit monoclonal antibodies (1:2000, EPR 15613-68, Abcam, Cambridge, MA, USA; 1:500, EPR20043, Abcam; 1:800, D8F12, CST, Danvers, MA, USA; respectively). After being incubated at 4°C for 16 h, the secondary antibodies were added. Next, the sections were counterstained with hematoxylin at room temperature for 3 min, dehydrated, and covered with a coverslip. According to the guideline protocol, positive controls were human skin tissue, human placenta tissue, and human urothelial carcinoma tissue for NECTIN-4, TROP-2, and HER2, respectively, and negative controls were UC tissues without primary antibodies.

NECTIN-4 expression was evaluated through the histochemical scoring system (H-score), which is defined as the product of intensity (score, 0–3), and percentage of stained cells (0–100). Then the specimens were classified as negative (0; H-score, 0–14), weak (1+; H-score, 15–99), moderate (2+; H-score, 100–199), and strong (3+; H-score, 200–300) (10). TROP-2 staining results were determined as follows: samples were deemed as positive if >10% tumor cells had membranous staining. Positive expression was scored as weak (+1), moderate (2+), and strong (3+). Tumors were classified as negative if <10% of tumor cells had membranous staining (26). For HER2, the staining scores were assessed according to the HER2 test guideline for breast cancer, and HER2 2+ and 3+ were defined as HER2-positive (16, 27).

### Statistics

SPSS software (version 26.0; SPSS, Inc., Chicago, IL, USA) was used for statistical analysis of all data, and P < 0.05 was considered as statistical significance. A Venn diagram was made through VENNY 2.1 (28).

## Results

**Figure 1:**
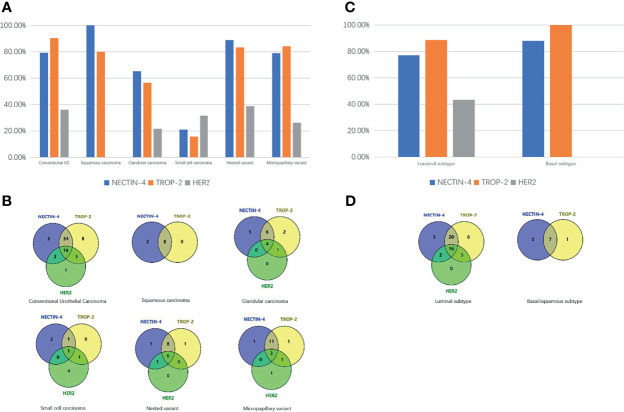
Expression differences of NECTIN-4, TROP-2, and HER2 in different pathologic types of UC. **(A)** Expression differences of HER2, TROP-2, and NECTIN-4 in conventional UC and histologic variants. **(B)** Venn diagram of positive expression distribution of NECTIN-4, TROP-2, and HER2 in conventional UC and histologic variants. **(C)** Expression differences of NECTIN-4, TROP-2, and HER2 in luminal and basal/squamous subtype. **(D)** Venn diagram of positive expression distribution of NECTIN-4, TROP-2, and HER2 in luminal and basal/squamous subtype.

The cohort included a total of 161 patients: 141 patients with bladder cancer and 20 patients with upper-tract urothelial carcinoma (ratio: 7.05:1); there were 126 men and 35 women (ratio 3.6: 1). The average age at diagnosis was 67.1 years (range 37 to 91 y) (**Table 1**). The samples were grouped based on the presence of divergent differentiation of pathological components into UC (n = 72); squamous carcinoma (n = 10); glandular (n = 23); small cell carcinoma (n = 19); micropapillary (n = 19); and nested (n = 18). Immunohistochemical results of NECTIN-4, TROP-2, and HER2 in different pathological types of UC are shown in **Figure 1** and **Supplementary 1**. Overall, the expressions of NECTIN-4, TROP-2, and HER2 were associated with histologic subtypes, but not to age, year, gender, tumor diameter, tumor location, and TNM grade (**Supplementary 2**).

**Table 1:** Clinicopathologic characteristics of patients enrolled.

Clinicopathological features	Conventional UC	Squamous carcinoma	Glandular carcinoma	Small cell carcinoma	Nested variants	Micropapillary variants
	(N = 72)	(N = 10)	(N = 23)	(N = 19)	(N = 18)	(N = 19)
**Age, years (SD, range)**	69.89 (8.14, 52–72)	70.5 (13.68, 40–84)	53.87 (11.93, 37–82)	72.05 (10.75, 52–91)	65.61 (7.48, 54–82)	67.26 (7.98, 53–82)
**Gender, n (%)**						
** M**	57 (79.2)	7 (70.0)	17 (73.9)	15 (78.9)	14 (77.8)	16 (84.2)
** F**	15 (20.8)	3 (30.0)	6 (26.1)	4 (21.1)	4 (22.2)	3 (15.8)
**Tumor diameters, cm (SD, range)**	3.27 (1.40, 1.0–9.0)	5.32 (3.15, 1.5–12.0)	3.71 (2.70, 1.0–14.0)	4.56 (3.16, 1.2–12.0)	3.76 (1.61, 1.5–7.0)	3.30 (1.54. 1.2–6.0)
**Tumor site (%)**						
** Bladder cancer**	61 (84.7)	9 (90.0)	21 (91.3)	17 (89.5)	17 (94.4)	16 (84.2)
** Upper-tract urothelial carcinoma**	11 (15.3)	1 (10.0)	2 (8.7)	2 (10.5)	1 (5.6)	3 (15.8)
**T-stage distribution (%)**						
** T2**	30 (41.7)	2 (20.0)	11 (47.8)	4 (21.1)	9 (50.0)	5 (26.3)
** T3**	29 (40.3)	5 (50.0)	11 (47.8)	14 (73.7)	8 (44.4)	6 (31.6)
** T4**	13 (18.1)	3 (30.0)	1 (4.3)	1 (5.3)	1 (5.6)	8 (42.1)
**Lymph node metastasis (%)**						
** N0**	61 (84.7)	6 (60.0)	18 (78.3)	14 (73.7)	14 (77.8)	10 (52.6)
** N1**	4 (5.6)	1 (10.0)	2 (8.7)	2 (10.5)	3 (16.8)	3 (15.8)
** N2**	7 (9.7)	3 (30.0)	3 (13)	3 (15.8)	1 (5.6)	6 (31.6)

### Urothelial Carcinoma

**Figure 2:**
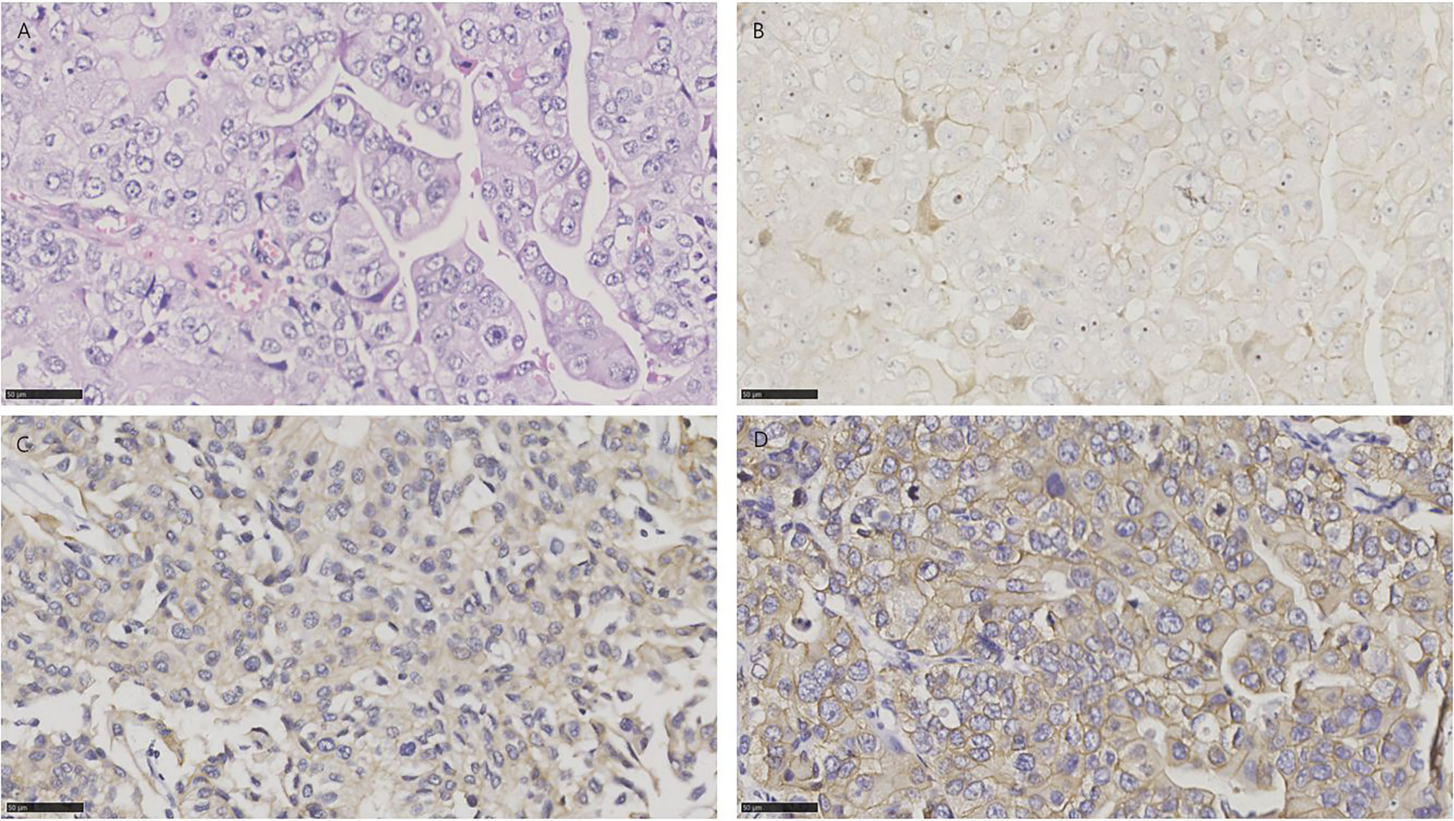
HE and immunohistochemistry for NECTIN-4, TROP-2, and HER2 in conventional urothelial carcinoma. Scale bar: 50 µm. **(A)** HE-stained section of conventional urothelial carcinoma. **(B)** Immunohistochemistry for NECTIN-4 in the same tumor showing moderate staining. **(C)** Immunohistochemistry for TROP-2 in the same tumor showing strong staining. **(D)** Immunohistochemistry for HER2 in the same tumor showing strong staining.

Overall, 57/72 (79.2%), 65/72 (90.3%), and 26/72 (36.1%) of UCs were positive for NECTIN-4, TROP-2, and HER2, respectively (**Figure 2**). 18 of 72 tissues (25.0%) were positive for all three targets, and 1 of 72 tissues (1.4%) was negative for the three. 52/72 (72.2%) were positive for both TROP-2 and NECTIN-4, 23/72 (31.9%) for both HER2 and TROP-2, and 20/72 (27.8%) for both HER2 and NECTIN-4 (**Figure 1B**). After being grouped by molecular classification, 53 luminal subtypes and 8 basal/squamous subtypes were obtained. The positive rates of NECTIN-4, TROP-2, and HER2 were 41/53 (77.35%), 47/53 (88.7%), and 23/53 (43.4%) in luminal subtypes, and 7/8 (87.5%), 8/8, and 0/6 in basal/squamous subtypes, respectively (**Figures 1C, D****)**.

### Squamous Carcinoma

**Figure 3:**
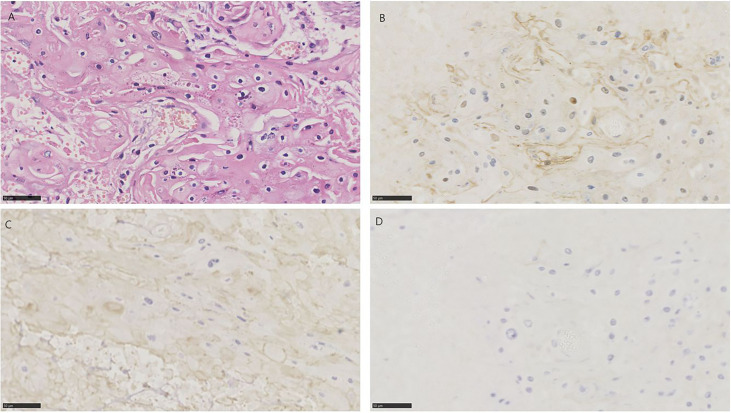
HE and immunohistochemistry for NECTIN-4, TROP-2, and HER2 in urothelial carcinoma with squamous differentiation. Scale bar: 50 µm. **(A)** HE-stained section of urothelial carcinoma with squamous differentiation. **(B)** Immunohistochemistry for NECTIN-4 in the same tumor showing moderate staining. **(C)** Immunohistochemistry for TROP-2 in the same tumor showing moderate staining. **(D)** Immunohistochemistry for HER2 in the same tumor showing negative.

There were 10 samples with at least 50% of the tumor displaying squamous differentiation, defined histologically by the presence of intracellular bridges or keratin (29). 10/10 (100%) for NECTIN-4, 8/10 (80%) for TROP-2, and 0/10 (0) for HER2 were positive, respectively (**Figures 1B**, **3**).

### Glandular Carcinoma

Glandular differentiation is histologically characterized by the presence of glandular spaces within the urothelial tumor (30). In all 23 specimens, 13/23 (56.5%) for NECTIN-4, 16/23 (69.5%) for TROP-2, and 5/23 (21.7%) for HER2 were positive, respectively. 4 of 23 tissues (17.4%) were positive for all three targets, and 5 of 23 tissues (21.7%) were negative for the three. 10/23 (43.5%) were positive for both TROP-2 and NECTIN-4, 5/23 (21.7%) for both HER2 and TROP-2, and 4/23 (17.4%) for both HER2 and NECTIN-4 (**Figure 1B**). The clinicopathologic characteristics of samples with three negative ADC targets and samples with at least one positive target are shown in **Supplementary 3**.

### Small Cell Carcinoma

**Figure 4:**
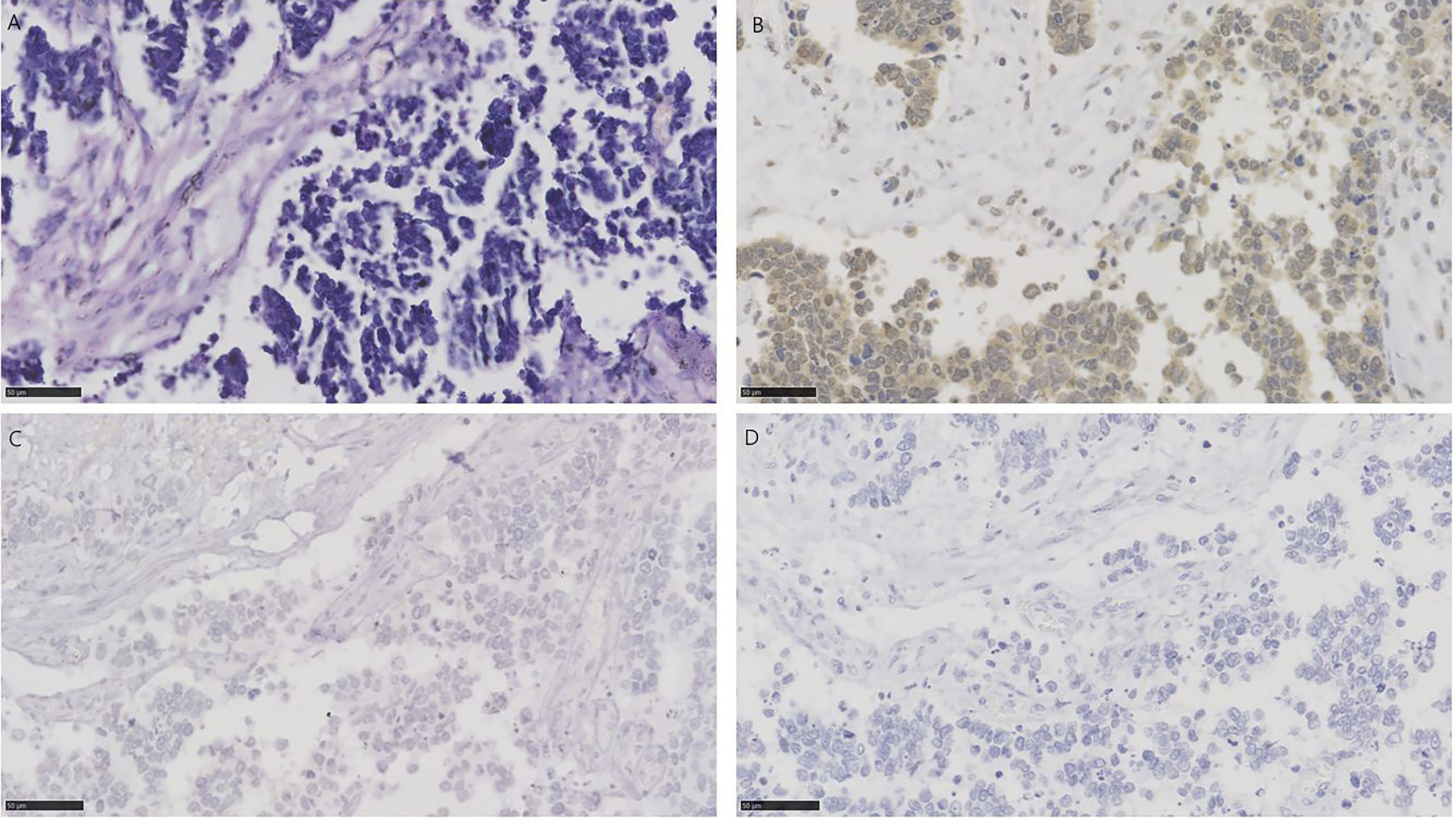
HE and immunohistochemistry for NECTIN-4, TROP-2, and HER2 in urothelial carcinoma with small cell carcinoma. Scale bar: 50 µm. **(A)** HE-stained section of urothelial carcinoma with small cell carcinoma. **(B)** Immunohistochemistry for NECTIN-4 in the same tumor showing moderate staining. **(C)** Immunohistochemistry for TROP-2 in the same tumor showing negative. **(D)** Immunohistochemistry for HER2 in the same tumor showing negative.

There were 19 specimens of small cell carcinoma, characterized by pathological features of spindle cells with scant cytoplasm and hyperchromatic nuclei with “salt and pepper” chromatin (31). 4/19 (21.1%) for NECTIN-4, 3/19 (15.8%) for TROP-2, and 6/19 (31.6%) for HER2 were positive, respectively (**Figure 4**). Only 1 of 19 tissues (5.3%) was positive for all three targets, and 10 of 19 tissues (52.6%) were negative for the three. 2/19 (10.5%) were positive for both TROP-2 and NECTIN-4, 2/19 (10.5%) for both HER2 and TROP-2, and 1/19 (5.3%) for both HER2 and NECTIN-4 (**Figure 1B**). The clinicopathologic characteristics of samples with three negative ADC targets and samples with at least one positive target are shown in **Supplementary 4**.

### Nested Variant

**Figure 5:**
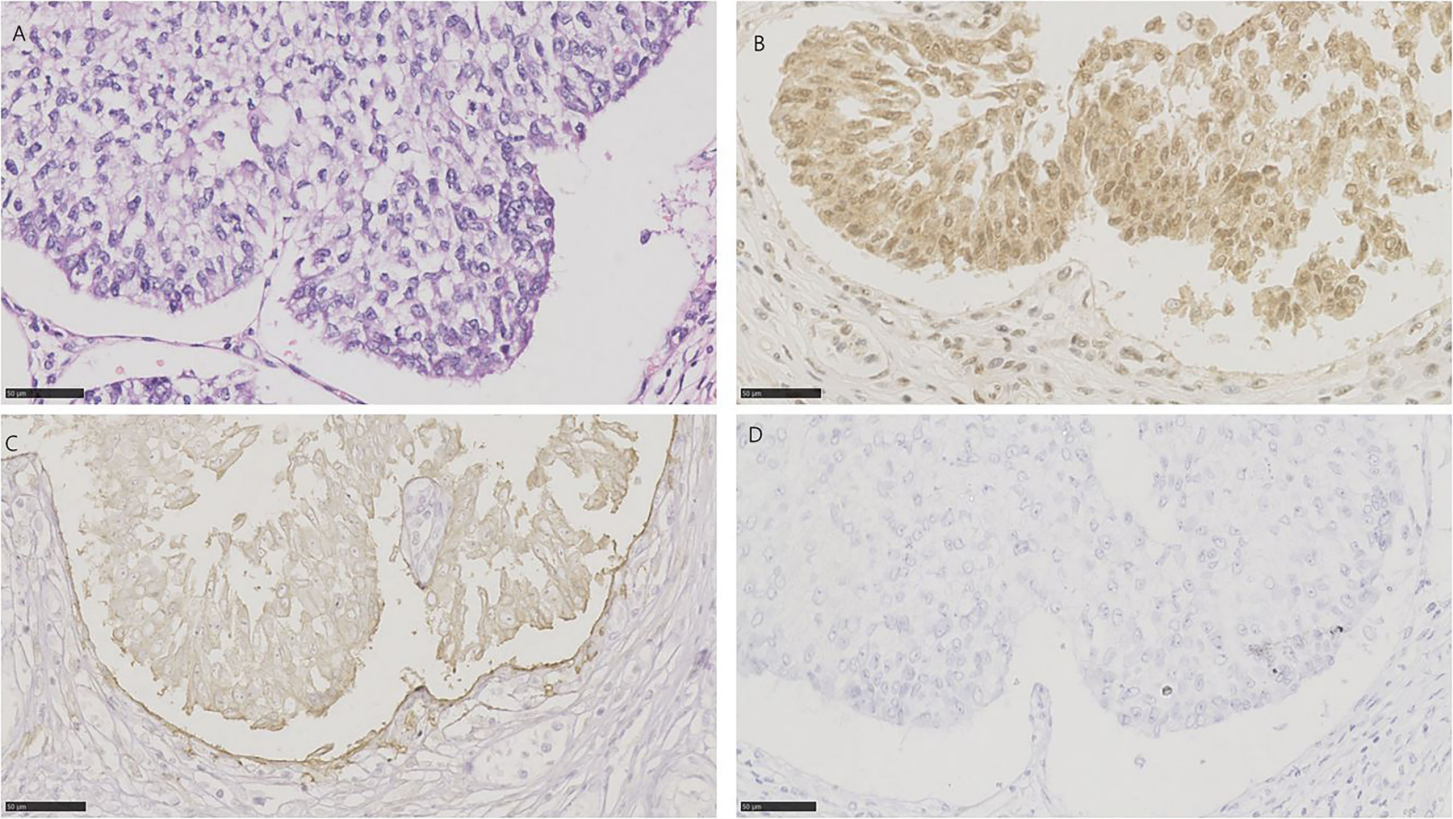
HE and immunohistochemistry for NECTIN-4, TROP-2, and HER2 in nested urothelial carcinoma. Scale bar: 50 µm. **(A)** HE-stained section of nested urothelial carcinoma. **(B)** Immunohistochemistry for NECTIN-4 in the same tumor showing strong staining. **(C)** Immunohistochemistry for TROP-2 in the same tumor showing moderate staining. **(D)** Immunohistochemistry for HER2 in the same tumor showing negative.

All 18 cases of nested urothelial carcinoma were detected, which are defined as bland nests of urothelial carcinoma [17]. 16/18 (88.9%) for NECTIN-4, 15/18 (83.3%) for TROP-2, and 7/18 (38.9%) for HER2 were positive, respectively (**Figure 5**). 6 of 18 tissues (33.3%) were positive for all three targets, and 1 of 18 tissues (5.6%) was negative for the three. 14/18 (77.8%) were positive for both TROP-2 and NECTIN-4, 6/18 (33.3%) for both HER2 and TROP-2, and 7/18 (38.9%) for both HER2 and NECTIN-4 (**Figure 1B**).

### Micropapillary Variant

**Figure 6:**
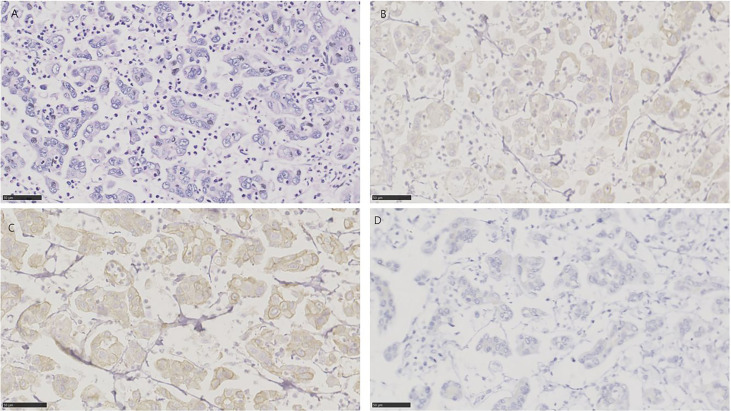
HE and immunohistochemistry for HER2, TROP-2, and NECTIN-4 in micropapillary urothelial carcinoma. Scale bar: 50 µm. **(A)** HE-stained section of micropapillary urothelial carcinoma. **(B)** Immunohistochemistry for NECTIN-4 in the same tumor showing weak staining. **(C)** Immunohistochemistry for TROP-2 in same tumor showing moderate staining. **(D)** Immunohistochemistry for HER2 in the same tumor showing negative.

A micropapillary variant was diagnosed by the presence of multiple nests of tumor within a single lacuna demonstrating small branching papillae or tufts without fibrovascular cores (32). In all 19 specimens, 15/19 (78.9%) for NECTIN-4, 16/19 (84.2%) for TROP-2, and 5/19 (26.3%) for HER2 were positive, respectively (**Figure 6**). 3 of 19 tissues (15.8%) were positive for all three targets, and 1 of 19 tissues (5.3%) was negative for the three. 14/19 (73.7%) were positive for both TROP-2 and NECTIN-4, 4/19 (21.1%) for both HER2 and TROP-2, and 3/19 (15.8%) for both HER2 and NECTIN-4 (**Figure 1B**).

## Discussion

Locally advanced and metastatic urothelial carcinoma often has a poor prognosis, with a median survival chemotherapy of approximately 13 to 15 months (33). The first-line treatment has been cisplatin-based cytotoxic chemotherapy for decades. In cisplatin-ineligible patients, carboplatin is an inferior alternative with a relatively worse objective response rate and median overall survival (34). The appearance of ADC represents a promising therapeutic approach for advanced patients or cisplatin-and carboplatin-ineligible patients. This novel technology targets surface proteins highly enriched in tumor to improve the delivery of cytotoxic molecules to tumor cells and reduce off-tumor toxicity. Three ADCs presented high activity in pretreated local advanced and metastatic UC, namely, EV, SG, and RC-48, targeting at the proteins of NECTIN-4, TROP-2, and HER2, respectively. Cells expressing these transmembrane proteins internalize them through endocytosis, resulting in the delivery and release of cytotoxic payload (35). So far, while EV and SG are FDA-approved in locally advanced and metastatic UC, RC-48 is still in clinical trials, although it has shown promising activity.

The expression of corresponding antigens on tumor is essential for appropriate functional delivery of the ADCs as it is the port of entry to tumor cells. However, except for the requirement for HER2-positive in RC-48 treatment, there is no requirement for testing the expressions of NECTIN-4 and TROP-2 in EV and SG treatment (16, 36, 37). In the phase 1 trial of EV, NECTIN-4 expression using an anti NECTIN-4 antibody clone was initially a protocol requirement, which was later removed due to high NECTIN-4 expression in most UC samples. However, these high expression rates are generally based on the UC. After being classified by histologic subtypes, the expression differences of these proteins were unknown.

So far, UC has been treated similarly regardless of its histologic subtypes, which mainly owes to the similar survival outcomes of most histologic variants (38). However, studies have shown that some histologic variants, such as squamous carcinoma and small cell carcinoma, have worse prognosis than UC and have a poorer response to standard chemotherapy (MVAC—methotrexate, vinblastine, doxorubicin, and cisplatin, or GC–gemcitabine and cisplatin) (39, 40), while other variants, such as urachal glandular carcinoma, have superior survival outcome and could be managed more conservatively (41). These suggest that UC should be managed selectively and individually according to its histologic features.

In this study, we confirmed that NECTIN-4 and TROP-2 were highly expressed in UC, while HER2 amplification was relatively low, which is consistent with the former clinical trials. Only 1.4% of conventional UCs were negative for the three targets, indicating that the majority of patients could benefit from the ADC therapy. Chu et al. reported that NECTIN-4 is enriched in luminal subtypes of muscle-invasive bladder cancer than in basal/squamous subtypes (42). In our study, the positive rate of NECTIN-4 in luminal subtypes and basal/squamous subtypes was 41/53 (77.35%) and 7/8 (87.5%), respectively. The difference was not statistically significant (P = 0.584). This may be due to the small number of basal/squamous subtypes. For histologic variants, we demonstrated that 0%, 21.7%, 52.6%, 5.6%, and 5.3% of squamous carcinoma, glandular carcinoma, small cell carcinoma, nested variant, and micropapillary variant were negative for the three targets, respectively, suggesting that therapeutic strategies for these subtypes should be made individually. HER2 expression was hardly in both histologic and molecular classifications of the squamous subtype, while TROP-2 and NECTIN-4 were expressed commonly, implying that these patients might benefit more from SG and NE rather than from RC-48. On the contrary, the expression levels of TROP-2 and NECTIN-4 decreased in small cell carcinoma, even being exceeded by that of HER2. Therefore, testing the expression of ADC targets should be considered before treatment and RC-48 may be the better choice for these patients. Furthermore, nested and micropapillary variants, whose survival outcomes and target distributions are similar to UC, are recommended to be managed as UC of the same stage (38).

In addition, these ADCs are unlikely to be cross-resistant as they carry different cytotoxic agents and target different antigens. Therefore, combination or sequence therapy may become the novel approach due to their controllable toxicity. Our study found respectively 72.2%, 90%, 43.5%, 77.8%, and 73.7% of UC, squamous carcinoma, glandular carcinoma, nested variant, and micropapillary variant expressing both TROP-2 and NECTIN-4, while 10.5% of small cell carcinoma, which may have some implications for combination and sequence therapies.

Currently, these ADCs are only recommended in second- or third-line therapy of local advanced and metastatic UC, so the expression situation of their targets could be to some extent ignored as the therapy approaches for the advanced stage are limited. Clinical trials about their application in neoadjuvant therapy of UC are in progress (43, 44). For the early stage, the expression situation of corresponding targets may play an important role in the choice of ACD therapy.

The primary limitation in our study is the lack of relation with therapy outcomes.

Our study only focuses on the head-to head comparison of expression differences of ADC targets, not involved in therapy efficacy. For histologic variants, the therapy efficacy dose depends not only on the expression of corresponding targets but also on the effect of conjugated cytotoxic agents. For example, SN-38, the conjugated cytotoxic payload of SG and the active component of irinotecan, showed efficacy only in colorectal, pancreatic, and lung cancer before (45). Therefore, it is not clear whether SG could achieve desired therapy outcomes in UC even if these histologic subtypes highly express TROP-2. However, the expression of corresponding targets could be seen as the basics of response to ADCs as it is the port of cellular entry for the cytotoxic drug component. Second, we detected target expression only through immunohistochemistry, which was not affirmed by other methods, such as FISH, RNA sequencing, or Western blot. Third, it was a retrospective study with a relatively small sample. These limitations should be addressed in future studies. In addition, samples in our study were from muscle-invasive tumors. Therefore, it is unclear to what extent this applies to metastatic samples.

## Conclusion

In summary, through a head-to-head comparison of expression differences of NECTIN-4, TROP-2, and HER2 in UC and its histologic variants, we provided evidence for therapeutic strategies for UC in an upcoming ADC ear. We demonstrate that the majority of UC and its histologic variant expressed at least one ADC target, suggesting that ADC is a candidate approach for UC therapy. However, different targets are expressed disparately in different histologic subtypes. Specific intervention strategies should be conducted individually according to the histologic subtypes.

## Data Availability Statement

The raw data supporting the conclusions of this article will be made available by the authors, without undue reservation.

## Author Contributions

QZ and YF contributed to the conception of the study. QL, ZL, QS, SH, and QH performed the experiment. YG, QL, and ZZ contributed significantly to analysis and manuscript preparation. YF and QL performed the data analyses and wrote the manuscript. WY and ZH helped perform the analysis with constructive discussions. All authors contributed to the article and approved the submitted version.

## Funding

This work was supported by grants from the National Natural Science Foundation of China to Q.Z. (No.82072826) and Special Project of “Group Medical Assistance Project” of Tibet Autonomous Region Health Committee (Grant No. XZ2019ZR-ZY16(Z)).

## Conflict of Interest

The authors declare that the research was conducted in the absence of any commercial or financial relationships that could be construed as a potential conflict of interest.

## Publisher’s Note

All claims expressed in this article are solely those of the authors and do not necessarily represent those of their affiliated organizations, or those of the publisher, the editors and the reviewers. Any product that may be evaluated in this article, or claim that may be made by its manufacturer, is not guaranteed or endorsed by the publisher.

## References

[1] Siegel RL Miller KD Jemal A. Cancer Statistics 2020. CA Cancer J Clin (2020) 70(1):7–30. doi: 10.3322/caac.21590 31912902

[2] Amin MB Smith SC Reuter VE Epstein JI Grignon DJ Hansel DE. Update for the Practicing Pathologist: The International Consultation On Urologic Disease-European Association of Urology Consultation on Bladder Cancer. Mod Pathol (2015) 28(5):612–30. doi: 10.1038/modpathol.2014.158 PMC5009623 25412849

[3] Ploeg M Aben KK Hulsbergen-van de Kaa CA Schoenberg MP Witjes JA Kiemeney LA. Clinical Epidemiology of Nonurothelial Bladder Cancer: Analysis of the Netherlands Cancer Registry. J Urol (2010) 183(3):915–20. doi: 10.1016/j.juro.2009.11.018 20083267

[4] Wucherpfennig S Rose M Maurer A Cassataro MA Seillier L Morsch R. Evaluation of Therapeutic Targets in Histological Subtypes of Bladder Cancer. Int J Mol Sci (2021) 22(21):11547. doi: 10.3390/ijms222111547 34768978 PMC8583926

[5] Singla N Fang D Su X Bao Z Cao Z Jafri SM. A Multi-Institutional Comparison of Clinicopathological Characteristics and Oncologic Outcomes of Upper Tract Urothelial Carcinoma in China and the United States. J Urol (2017) 197(5):1208–13. doi: 10.1016/j.juro.2016.11.094 27887951

[6] NCCN Guidelines Version 6.2020 Bladder Cancer. Available at: https://www.nccn.org/professionals/physician_gls/pdf/bladder.pdf (Accessed july 16).

[7] Sarfaty M Rosenberg JE. Antibody-Drug Conjugates in Urothelial Carcinomas. Curr Oncol Rep (2020) 22(2):13. doi: 10.1007/s11912-020-0879-y 32008109

[8] Goldenberg DM Sharkey RM. Sacituzumab Govitecan, a Novel, Third-Generation, Antibody-Drug Conjugate (ADC) for Cancer Therapy. Expert Opin Biol Ther (2020) 20(8):871–85. doi: 10.1080/14712598.2020.1757067 32301634

[9] Samanta D Almo SC. Nectin Family of Cell-Adhesion Molecules: Structural and Molecular Aspects of Function and Specificity. Cell Mol Life Sci (2015) 72(4):645–58. doi: 10.1007/s00018-014-1763-4 PMC11113404 25326769

[10] Challita-Eid PM Satpayev D Yang P An Z Morrison K Shostak Y. Enfortumab Vedotin Antibody-Drug Conjugate Targeting Nectin-4 Is a Highly Potent Therapeutic Agent in Multiple Preclinical Cancer Models. Cancer Res (2016) 76(10):3003–13. doi: 10.1158/0008-5472.Can-15-1313 27013195

[11] Cubas R Zhang S Li M Chen C Yao Q. Trop2 Expression Contributes to Tumor Pathogenesis by Activating the ERK MAPK Pathway. Mol Cancer (2010) 9:253. doi: 10.1186/1476-4598-9-253 20858281 PMC2946292

[12] Avellini C Licini C Lazzarini R Gesuita R Guerra E Tossetta G. The Trophoblast Cell Surface Antigen 2 and miR-125b Axis in Urothelial Bladder Cancer. Oncotarget (2017) 8(35):58642–53. doi: 10.18632/oncotarget.17407 PMC5601681 28938585

[13] Goldenberg DM Sharkey RM. Antibody-Drug Conjugates Targeting TROP-2 and Incorporating SN-38: A Case Study of Anti-TROP-2 Sacituzumab Govitecan. MAbs (2019) 11(6):987–95. doi: 10.1080/19420862.2019.1632115 PMC6748572 31208270

[14] Fleischmann A Rotzer D Seiler R Studer UE Thalmann GN. Her2 Amplification Is Significantly More Frequent in Lymph Node Metastases From Urothelial Bladder Cancer Than in the Primary Tumours. Eur Urol (2011) 60(2):350–7. doi: 10.1016/j.eururo.2011.05.035 21640482

[15] Jimenez RE Hussain M Bianco FJ Jr. Vaishampayan U Tabazcka P Sakr WA. Her-2/Neu Overexpression in Muscle-Invasive Urothelial Carcinoma of the Bladder: Prognostic Significance and Comparative Analysis in Primary and Metastatic Tumors. Clin Cancer Res (2001) 7(8):2440–7. 11489824

[16] Sheng X Yan X Wang L Shi Y Yao X Luo H. Open-Label, Multicenter, Phase II Study of RC48-ADC, A HER2-Targeting Antibody-Drug Conjugate, in Patients With Locally Advanced or Metastatic Urothelial Carcinoma. Clin Cancer Res (2021) 27(1):43–51. doi: 10.1158/1078-0432.Ccr-20-2488 33109737

[17] Zhang B Yu W Feng X Zhao Z Fan Y Meng Y. Prognostic Significance of PD-L1 Expression on Tumor Cells and Tumor-Infiltrating Mononuclear Cells in Upper Tract Urothelial Carcinoma. Med Oncol (2017) 34(5):94. doi: 10.1007/s12032-017-0941-2 28409437

[18] Liu Z Meng Y Cao Y Chen Y Fan Y Li S. Expression and Prognostic Value of PD-L1 in Non-Schistosoma-Associated Urinary Bladder Squamous Cell Carcinoma. Transl Androl Urol (2020) 9(2):428–36. doi: 10.21037/tau.2020.02.12 PMC7215043 32420148

[19] Ghate K Amir E Kuksis M Hernandez-Barajas D Rodriguez-Romo L Booth CM. PD-L1 Expression and Clinical Outcomes in Patients With Advanced Urothelial Carcinoma Treated With Checkpoint Inhibitors: A Meta-Analysis. Cancer Treat Rev (2019) 76:51–6. doi: 10.1016/j.ctrv.2019.05.002 31125908

[20] Hoffman-Censits JH Lombardo KA Parimi V Kamanda S Choi W Hahn NM. Expression of Nectin-4 in Bladder Urothelial Carcinoma, in Morphologic Variants, and Nonurothelial Histotypes. Appl Immunohistochem Mol Morphol (2021) 29(8):619–25. doi: 10.1097/pai.0000000000000938 PMC8429050 33901032

[21] Faltas B Goldenberg DM Ocean AJ Govindan SV Wilhelm F Sharkey RM. Sacituzumab Govitecan, a Novel Antibody–Drug Conjugate, in Patients With Metastatic Platinum-Resistant Urothelial Carcinoma. Clin Genitourin Cancer (2016) 14(1):e75–9. doi: 10.1016/j.clgc.2015.10.002 26541586

[22] Ulbright TM Amin MB Balzer B Berney DM Epstein JI Guo C. WHO Classification of Tumours of the Urinary System and Male Genital Organs. Lyon, France: International Agency for Research on Cancer (2016).

[23] Sobin LH Gospodarowicz MK Wittekind C. TNM Classification of Malignant Tumours, 7th Edition . Chichester, UK: Wiley-Blackwell (2009).

[24] Guo CC Bondaruk J Yao H Wang Z Zhang L Lee S. Assessment of Luminal and Basal Phenotypes in Bladder Cancer. Sci Rep (2020) 10(1):9743. doi: 10.1038/s41598-020-66747-7 32546765 PMC7298008

[25] Dadhania V Zhang M Zhang L Bondaruk J Majewski T Siefker-Radtke A. Meta-Analysis of the Luminal and Basal Subtypes of Bladder Cancer and the Identification of Signature Immunohistochemical Markers for Clinical Use. EBioMedicine (2016) 12:105–17. doi: 10.1016/j.ebiom.2016.08.036 PMC5078592 27612592

[26] Bednova O Leyton JV. Targeted Molecular Therapeutics for Bladder Cancer-A New Option Beyond the Mixed Fortunes of Immune Checkpoint Inhibitors? Int J Mol Sci (2020) 21(19):7268. doi: 10.3390/ijms21197268 PMC7582582 33019653

[27] Wolff AC Hammond ME Hicks DG Dowsett M McShane LM Allison KH. Recommendations for Human Epidermal Growth Factor Receptor 2 Testing in Breast Cancer: American Society of Clinical Oncology/College of American Pathologists Clinical Practice Guideline Update. J Clin Oncol (2013) 31(31):3997–4013. doi: 10.1200/JCO.2013.50.9984 24101045

[28] Oliveros JC. Venny. An Interactive Tool for Comparing Lists With Venn's Diagrams (2007-2015). Available at: https://bioinfogp.cnb.csic.es/tools/venny/index.html.

[29] Lopez-Beltran A Henriques V Montironi R Cimadamore A Raspollini MR Cheng L. Variants and New Entities of Bladder Cancer. Histopathology (2019) 74(1):77–96. doi: 10.1111/his.13752 30565299

[30] Sfakianos JP Gul Z Shariat SF Matin SF Daneshmand S Plimack E. Genetic Differences Between Bladder and Upper Urinary Tract Carcinoma: Implications for Therapy. Eur Urol Oncol (2021) 4(2):170–9. doi: 10.1016/j.euo.2020.12.007 33386276

[31] Fahed E Hansel DE Raghavan D Quinn DI Dorff TB. Small Cell Bladder Cancer: Biology and Management. Semin Oncol (2012) 39(5):615–8. doi: 10.1053/j.seminoncol.2012.08.009 23040258

[32] Hui Y Lombardo KA Quddus MR Matoso A. Cell Polarity Reversal Distinguishes True Micropapillary Growth From Retraction Artifact in Invasive Urothelial Carcinoma. Appl Immunohistochem Mol Morphol (2018) 26(1):e1–6. doi: 10.1097/PAI.0000000000000566 28800010

[33] Nakagawa T Taguchi S Kanatani A Kawai T Ikeda M Urakami S. Oncologic Outcome of Metastasectomy for Urothelial Carcinoma: Who Is the Best Candidate? Ann Surg Oncol (2017) 24(9):2794–800. doi: 10.1245/s10434-017-5970-8 28687875

[34] Freshwater T Li H Valiathan C Li M Perini R Bracco OL. Systematic Literature Review and Meta-Analysis of Response to First-Line Therapies for Advanced/Metastatic Urothelial Cancer Patients Who Are Cisplatin Ineligible. Am J Clin Oncol (2019) 42(10):802–9. doi: 10.1097/COC.0000000000000585 31503064

[35] Chau CH Steeg PS Figg WD. Antibody-Drug Conjugates for Cancer. Lancet (2019) 394(10200):793–804. doi: 10.1016/S0140-6736(19)31774-X 31478503

[36] Powles T Rosenberg JE Sonpavde GP Loriot Y DurÃ¡n I Lee JL. Enfortumab Vedotin in Previously Treated Advanced Urothelial Carcinoma. N Engl J Med (2021) 384(12):1125–35. doi: 10.1056/NEJMoa2035807 PMC8450892 33577729

[37] Tagawa ST Balar AV Petrylak DP Kalebasty AR Loriot Y FlÃ©chon A. TROPHY-U-01: A Phase II Open-Label Study of Sacituzumab Govitecan in Patients With Metastatic Urothelial Carcinoma Progressing After Platinum-Based Chemotherapy and Checkpoint Inhibitors. J Clin Oncol (2021) 39(22):2474–85. doi: 10.1200/jco.20.03489 PMC8315301 33929895

[38] Lobo N Shariat SF Guo CC Fernandez MI Kassouf W Choudhury A. What Is the Significance of Variant Histology in Urothelial Carcinoma? Eur Urol Focus (2020) 6(4):653–63. doi: 10.1016/j.euf.2019.09.003 31530497

[39] Minato A Fujimoto N Kubo T. Squamous Differentiation Predicts Poor Response to Cisplatin-Based Chemotherapy and Unfavorable Prognosis in Urothelial Carcinoma of the Urinary Bladder. Clin Genitourin Cancer (2017) 15(6):e1063–7. doi: 10.1016/j.clgc.2017.07.008 28803791

[40] Lim JH Sundar S. Prognosis of Early Stage Small Cell Bladder Cancer Is Not Always Dismal. ESMO Open (2019) 4(6):e000559. doi: 10.1136/esmoopen-2019-000559 31798978 PMC6863661

[41] Dutta R Abdelhalim A Martin JW Vernez SL Faltas B Lotan Y. Effect of Tumor Location on Survival in Urinary Bladder Adenocarcinoma: A Population-Based Analysis. Urol Oncol (2016) 34(12):531.e1–6. doi: 10.1016/j.urolonc.2016.06.009 27427223

[42] Chu CE Sjostrom M Egusa EA Gibb EA Badura ML Zhu J. Heterogeneity in NECTIN4 Expression Across Molecular Subtypes of Urothelial Cancer Mediates Sensitivity to Enfortumab Vedotin. Clin Cancer Res (2021) 27(18):5123–30. doi: 10.1158/1078-0432.Ccr-20-4175 PMC8634828 34108177

[43] Fang D Kitamura H. Cancer Stem Cells and Epithelial-Mesenchymal Transition in Urothelial Carcinoma: Possible Pathways and Potential Therapeutic Approaches. Int J Urol (2018) 25(1):7–17. doi: 10.1111/iju.13404 28697535

[44] US National Library of Medicine. ClinicalTrials.gov. Available at: https://clinicaltrials.gov/ct2/show/NCT03924895.

[45] de Man FM Goey AKL van Schaik RHN Mathijssen RHJ Bins S. Individualization of Irinotecan Treatment: A Review of Pharmacokinetics, Pharmacodynamics, and Pharmacogenetics. Clin Pharmacokinet (2018) 57(10):1229–54. doi: 10.1007/s40262-018-0644-7 PMC6132501 29520731

