# Supplementary material for: Head-to-Head Comparison of the Expression Differences of NECTIN-4, TROP-2, and HER2 in Urothelial Carcinoma and Its Histologic Variants
Source: Front Oncol. 2022 Apr 19;12:858865. doi: 10.3389/fonc.2022.858865 (PMC9063095; doi:10.3389/fonc.2022.858865)
Supplement: Supplementary file 1 [file Table_1.docx]

Supplementary Material

**Supplement 1.** Immunohistochemical results of NECTIN-4, TROP-2 and HER2 in histological subtypes of UC.

| **Histological Subtype** | | | | | | | |
| --- | --- | --- | --- | --- | --- | --- | --- |
| **Target** | **Staining intensity** | **Conventional UC** | **Squamous carcinoma** | **Glandular carcinoma** | **Small cell carcinoma** | **Nested variants** | **Micropapillary variants** |
| **NECTIN-4** | 0 | 15 | 0 | 8 | 15 | 2 | 4 |
|  | 1 | 10 | 3 | 8 | 0 | 8 | 10 |
|  | 2 | 23 | 5 | 6 | 3 | 7 | 4 |
|  | 3 | 24 | 2 | 1 | 1 | 1 | 1 |
| **TROP-2** | 0 | 7 | 2 | 7 | 16 | 3 | 3 |
|  | 1 | 12 | 2 | 2 | 0 | 5 | 4 |
|  | 2 | 26 | 3 | 8 | 2 | 6 | 6 |
|  | 3 | 27 | 3 | 6 | 1 | 4 | 6 |
| **HER2** | 0 | 30 | 10 | 16 | 12 | 11 | 12 |
|  | 1 | 16 | 0 | 2 | 1 | 0 | 2 |
|  | 2 | 12 | 0 | 3 | 4 | 2 | 2 |
|  | 3 | 14 | 0 | 2 | 2 | 5 | 3 |

**Supplement 2**. Logistic regression analyses for the expression situation of NECTIN-4, TROP-2 and HER2

| **Clinicopathological Features** | **NECTIN-4** | | **TROP-2** | | **HER2** | |
| --- | --- | --- | --- | --- | --- | --- |
|  | OR (95% CI) | P Value | OR (95% CI) | P Value | OR (95% CI) | P Value |
| **Age** | 1.004(0.955-1.055) | 0.879 | 0.995(0.948-1.044) | 0.830 | 0.985(0.944-1.029) | 0.503 |
| **Gender** |  |  |  |  |  |  |
| **Male** | 1 |  | 1 |  | 1 |  |
| **Female** | 0.285（0.078-1.034） | 0.056 | 0.827(0.267-2.557) | 0.741 | 1.307(0.529-3.225) | 0.562 |
| **Tumor diameters** | 0.881(0.691-1.124) | 0.309 | 0.172 | 0.172 | 0.856(0.674-1.088) | 0.204 |
| **Tumor site** |  |  |  |  |  |  |
| **Bladder cancer** | 1 |  | 1 |  | 1 |  |
| **Upper-tract urothelial carcinoma** | 1.445（0.316-6.593）） | 0.635 | 0.952 (0.225-4.020) | 0.947 | 0.642(0.189-2.177) | 0.476 |
| **T Stage distribution** |  |  |  |  |  |  |
| **T2** | 1 |  | 1 |  | 1 |  |
| **T3** | 0.652（0.160-2.661）） | 0.551 | 0.705(0.115-4.318) | 0.705 | 0.692(0.210-2.280) | 0.545 |
| **T4** | 3.224（0.785-13.239） | 0.104 | 0.340(0.066-1.746) | 0.196 | 0.514(0.164-1.606) | 0.252 |
| **Lymph node metastasis** |  |  |  |  |  |  |
| **N1** | 1 |  | 1 |  | 1 |  |
| **N2** | 2.059(0.462-9.163) | 0.343 | 0.664(0.147-2.997) | 0.595 | 1.122(0.308-4.092) | 0.861 |
| **N3** | 0.562(0.093-3.394) | 0.530 | 0.229(0.038-1.382) | 0.108 | 1.358(0.279-6.603) | 0.705 |
| **Histologic classification** |  |  |  |  |  |  |
| **Conventional UC** | 1 |  | 1 |  | 1 |  |
| **Squamous carcinoma** |  |  | 0.840(0.093-7.611) | 0.877 |  |  |
| **Glandular carcinoma** | 0.174(0.025-1.200) | 0.076 | 0.185(0.033-1.034) | 0.055 | 0.355(0.078-1.608) | 0.179 |
| **Small cell carcinoma** | 0.012(0.001-0.107) | <0.03 | 0.035(0.005-0.224) | <0.03 | 0.976(0.234-4.076) | 0.974 |
| **Nested variants** | 0.479(0.058-3.947) | 0.494 | 0.671(0.092-4.920) | 0.695 | 0.457(0.100-2.095) | 0.313 |
| **Micropapillary variants** | 0.343(0.057-2.049) | 0.240 | 1.375(0.278-6.788) | 0.696 | 0.880(0.283-2.735) | 0.825 |

**Supplement 3**. Clinicopathologic characteristics of glandular carcinoma with three negative ADC targets and glandular carcinoma with at least one positive target.

| **Clinicopathological Features** | **Glandular carcinoma with three negative ADC targets** | **Glandular carcinoma with at least one positive target** | **P-value** |
| --- | --- | --- | --- |
|  | **N=5** | **N=18** |  |
| **Age, years (SD, range)** | 56.80(10.76, 38-65) | 53.06(12.40, 37-82) | 0.547 |
| **Gender, n (%)** |  |  | 0.133 |
| **M** | 5 (100) | 12 (66.7) |  |
| **F** | 0 | 6 (33.3) |  |
| **Tumor diameters, cm (SD, range)** | 6.24 (5.04, 1.0-14.0) | 3.01 (1.01, 1.5-5.0) | 0.014 |
| **Tumor site (%)** |  |  | 0.435 |
| **Bladder cancer** | 5 (100.0) | 16 (88.9) |  |
| **Upper-tract urothelial carcinoma** | 0 | 2 (11.1) |  |
| **T Stage distribution (%)** |  |  | 0.757 |
| **T2** | 2 (40.0) | 9 (50.0) |  |
| **T3** | 3 (60.0) | 8 (44.4) |  |
| **T4** | 0 | 1 (5.6) |  |
| **Lymph node metastasis (%)** |  |  | 0.058 |
| **N0** | 2 (40.0) | 16 (88.9) |  |
| **N1** | 1 (20.0) | 1 (5.6) |  |
| **N2** | 2 (40.0) | 1 (5.6) |  |

**Supplement 4**. Clinicopathologic characteristics of small cell carcinoma with three negative ADC targets and small cell carcinoma with at least one positive target.

| **Clinicopathological Features** | **Small cell carcinoma with three negative ADC targets** | **Small cell carcinoma with at least one positive target** | **P value** |
| --- | --- | --- | --- |
|  | **N=10** | **N=9** |  |
| **Age, years (SD, range)** | 72.90 (11.10, 52-85) | 71.11 (10.93, 60-91) | 0.728 |
| **Gender, n (%)** |  |  | 0.313 |
| **M** | 7 (70.0) | 8 (88.9) |  |
| **F** | 3(30.0) | 1 (11.1) |  |
| **Tumor diameters, cm (SD, range)** | 4.59 (3.08, 1.9-10.0) | 4.53 (3.43, 1.2-12.0) | 0.970 |
| **Tumor site (%)** |  |  | 0.156 |
| **Bladder cancer** | 8 (80.0) | 9 (100.0) |  |
| **Upper-tract urothelial carcinoma** | 2 (20.0) | 0 |  |
| **T Stage distribution (%)** |  |  | 0.047 |
| **T2** | 4 (40.0) | 0 |  |
| **T3** | 5 (50.0) | 9 (100.0) |  |
| **T4** | 1 (10.0) | 0 |  |
| **Lymph node metastasis (%)** |  |  | 0.276 |
| **N0** | 6 (60.0) | 8 (88.9) |  |
| **N1** | 2 (20.0) | 0 |  |
| **N2** | 2 (20.0) | 1 (11.1) |  |
